# Malignant Extrapleural Solitary Fibrous Tumor

**DOI:** 10.7759/cureus.43750

**Published:** 2023-08-19

**Authors:** Boluwatito T Abraham, Prithvi Balaji, Jae Woo Lee, Wendy Verola, John T Williams

**Affiliations:** 1 General Surgery, Trinity School of Medicine, Warner Robins, USA; 2 General Surgery, Coliseum Medical Centers, Macon, USA

**Keywords:** extrapleural, cd34, nab2-stat6, malignant, solitary fibrous tumor (sft)

## Abstract

Solitary fibrous tumors (SFTs) are rare spindle cell neoplasms of mesenchymal origin that are most commonly found in the pleura, although they have also been documented in extrapleural locations. SFTs affect males and females in equal distribution, and they typically occur between the fourth and seventh decades of life. Since SFTs are usually benign and asymptomatic, the majority of them are discovered incidentally on computed tomography (CT) or magnetic resonance imaging (MRI) imaging, unless they grow to a size that causes mass effect symptoms on other organs. Nonetheless, imaging is not sufficient to diagnose an SFT, and therefore, biopsy is recommended for further analysis. Advances in immunohistochemistry and molecular diagnostics have identified CD34 and NAB2-STAT6, respectively, as the most consistent markers for SFTs. The risk of SFT metastasis can be determined through the use of a four-variable risk-stratification model developed by Demicco et al., which is based upon the risk factors of patient age, tumor size, mitotic count per 10 high-power fields, and the degree of tumor necrosis. The management of SFTs involves a wide surgical resection of the tumor while preserving surrounding organs and structures. Post-operative surveillance involves imaging the primary tumor site for up to five years due to the risk of local recurrence. At this time, neither radiation therapy nor chemotherapy after resection have yet to show benefit, and therefore, they are not currently recommended. This case report discusses the management of a 68-year-old woman who was diagnosed with a malignant extrapleural SFT in her right medial upper thigh.

## Introduction

Solitary ﬁbrous tumors (SFTs) are rare spindle cell neoplasms of mesenchymal origin, which were initially identiﬁed in the pleura by Klemperer and Rabin in 1931 [1]. SFTs are most commonly found in intrathoracic locations, particularly the pleura, lung parenchyma, anterior mediastinum, diaphragm, and pericardium [1,2]. The abdomen is the second most common location, particularly intraperitoneally, retroperitoneally in the kidneys, and within the pelvis [1]. Lastly, SFTs are also known to arise within the sinonasal tract, oral cavity, epiglottis, salivary glands, orbit, meninges, thyroid, breasts, and extremities [1,2].

SFTs affect males and females in equal distribution and typically occur in adults between their fourth and seventh decades of life, with a median age of 50 years old [1,3]. They account for less than 2% of all soft tissue masses, of which 78-88% of SFTs are benign while 12-22% of SFTs are malignant [4].

Here, we present the case of a 68-year-old woman with a malignant extrapleural SFT in her right medial upper thigh. Pathology report is discussed along with clinical symptoms, past medical history, differential diagnosis, and treatment for the patient.

## Case presentation

A 68-year-old Caucasian female presented to the emergency department with a complaint of a new-onset palpable knot in her right medial upper thigh, which was deep, warm, and tender to the touch. The patient reported no trauma or interventional procedures to the area, and she also denied any redness, drainage, or fever. A Doppler ultrasound showed an anechoic circumscribed collection at a size of 9.2 x 3.9 x 2.7 cm, which contained a free-floating heterogenous echogenic material, located in the deep subcutaneous soft tissues superﬁcial to the femoral vasculature. The use of color-flow imaging only showed minimal color flow with arterialized waveforms at the margins of the collection; however, this was thought to represent transmitted pulses from adjacent arterial vasculature in the thigh. Furthermore, no definitive connection to the underlying femoral vasculature could be demonstrated. The lesion was compressible under transducer pressure, but it elicited a pain response from the patient. The emergency department then diagnosed the patient with a soft tissue abscess, prescribed her clindamycin, and advised her to follow-up with general surgery.

When the patient presented to the general surgery oﬃce, she denied any signiﬁcant changes to her symptoms of a deep, warm, and painful mass in her right thigh. Furthermore, she continued to deny any redness, drainage, or fever. Her past medical history was signiﬁcant for immunoglobulin A (IgA) deﬁciency, paroxysmal nocturnal hemoglobinuria, chronic kidney disease, polycystic kidney disease, hypertension, hyperlipidemia, myelodysplastic syndrome, and hysterectomy. Lastly, the patient denied both tobacco use and alcohol consumption.

On examination, the patient was alert and oriented. She was without tachypnea, tachycardia, or any other abnormalities on cardiopulmonary auscultation. Examination of extremities was unremarkable, with the exception of tenderness in her right medial upper thigh. An attempt to aspirate the lesion with ultrasound guidance was unsuccessful and resulted in a miniscule amount of thick fluid. Therefore, surgery was scheduled for incision and drainage.

**Figure 1:**
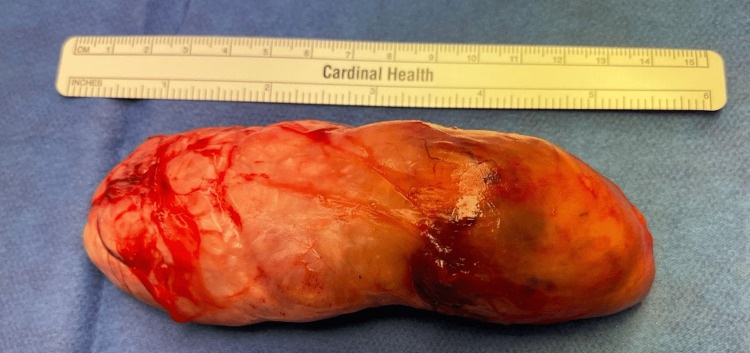
Solitary fibrous tumor (12 x 4.5 cm).

The patient was taken to the operating room and placed under general anesthesia, and her right medial thigh was prepped and draped. Timeout then followed. First, a local cut was made down to the mass, and incision and drainage of the mass were attempted unsuccessfully. A decision was then made to dissect out the edges of the mass in order to extract it from below the fascia of the thigh. The entire intact capsule of 12 x 4.5 cm was removed, as shown in Figure 1, and then sent to pathology. Although it had some attachments, they were neither vascular nor neural structures. The incision was closed in layers of 3-0 vicryl and 3-0 nylon sutures, after which dressing was applied. The patient was then taken to the recovery room in stable condition, and her postoperative recovery period has been unremarkable and without complications.

**Figure 2:**
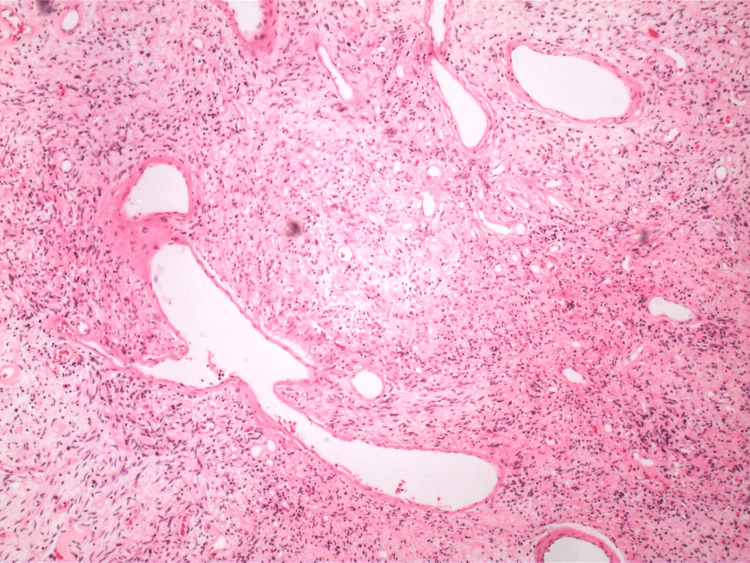
40x magnification of solitary fibrous tumor demonstrating staghorn vasculature.

**Figure 3:**
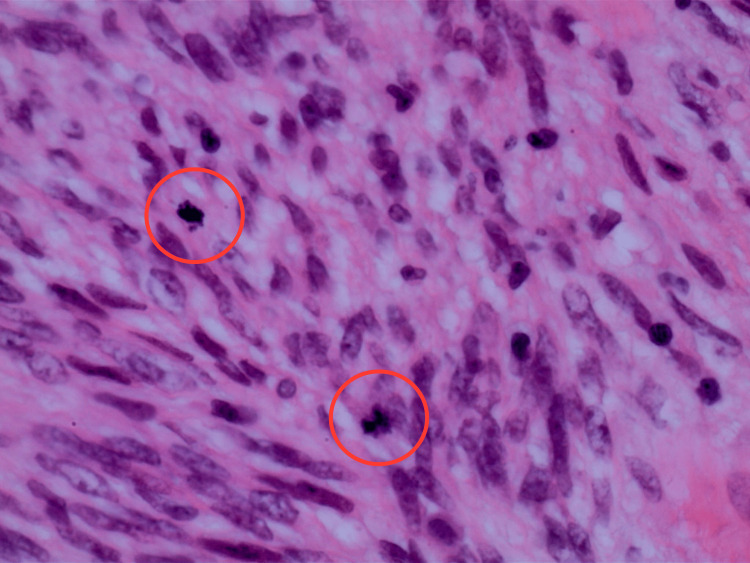
400x magnification of solitary fibrous tumor demonstrating 2 mitoses per 10 high-power fields (circled in red).

**Figure 4:**
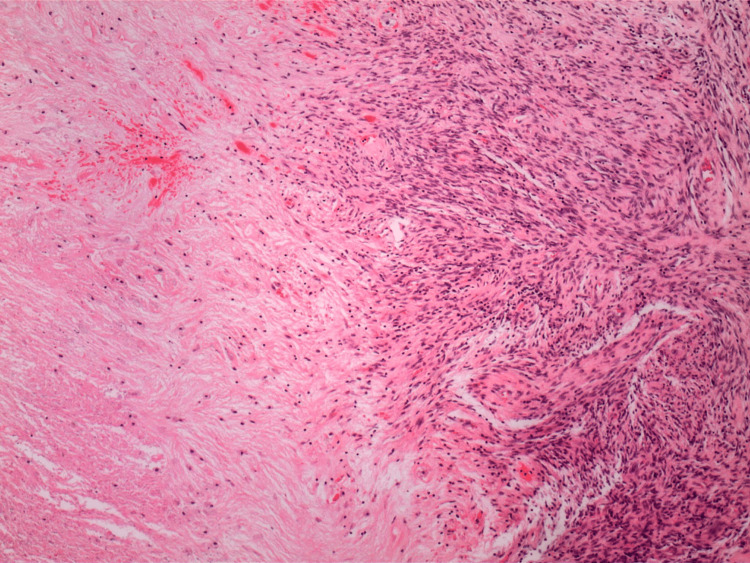
40x magnification of solitary fibrous tumor demonstrating tumor necrosis.

**Figure 5:**
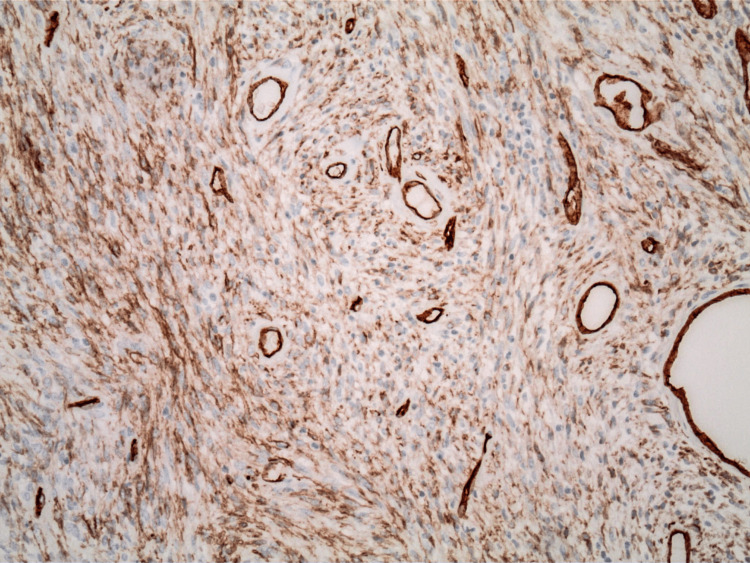
100x magnification of solitary fibrous tumor with immunohistochemical staining that is positive for CD34.

**Figure 6:**
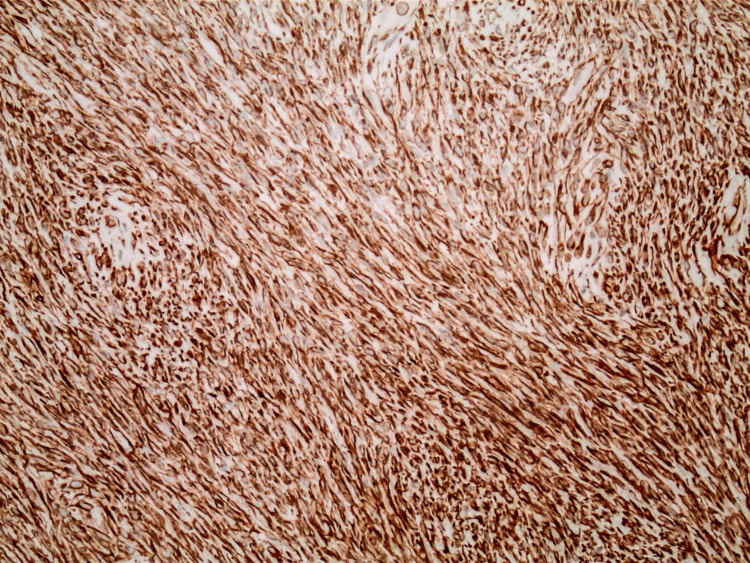
100x magnification of solitary fibrous tumor with immunohistochemical staining that is positive for vimentin.

**Figure 7:**
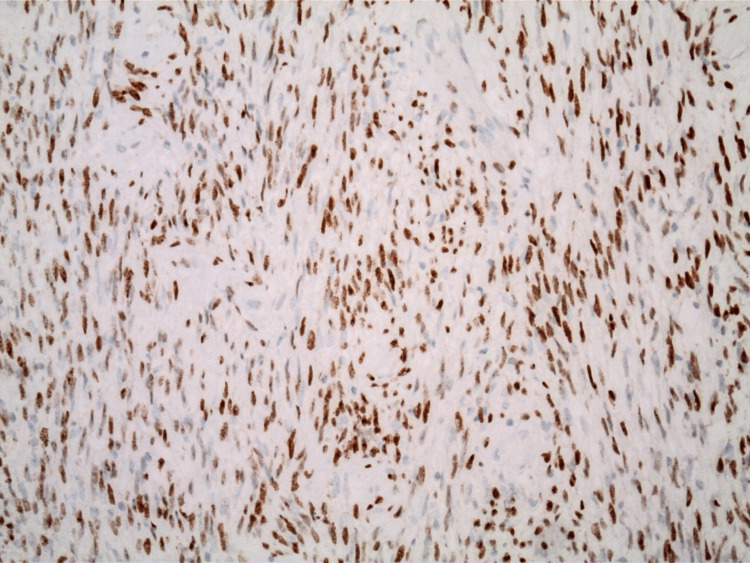
100x magnification of solitary fibrous tumor with immunohistochemical staining that is positive for STAT6.

**Figure 8:**
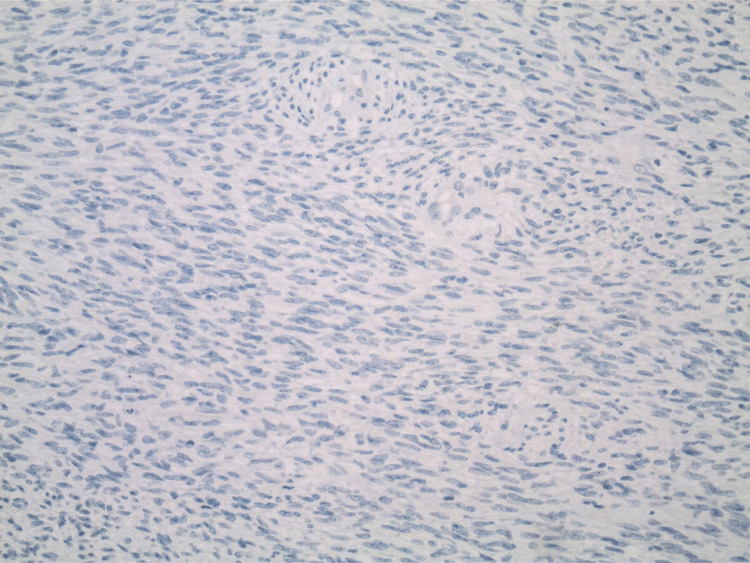
100x magnification of solitary fibrous tumor with immunohistochemical staining that is negative for SOX-10.

**Figure 9:**
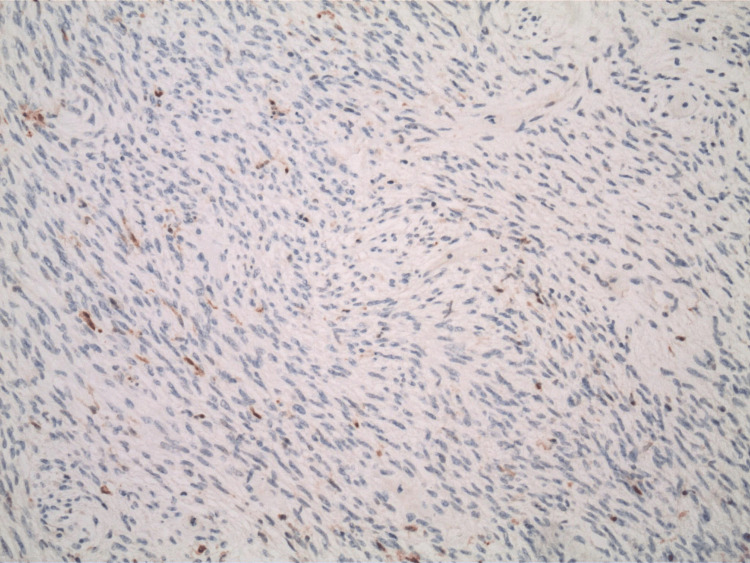
100x magnification of solitary fibrous tumor with immunohistochemical staining that is negative for S-100.

**Figure 10:**
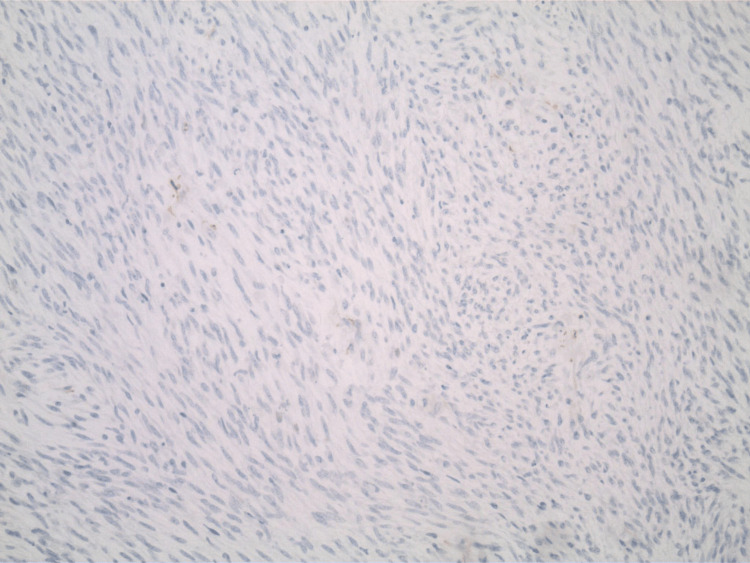
100x magnification of solitary fibrous tumor with immunohistochemical staining that is negative for SMA.

**Figure 11:**
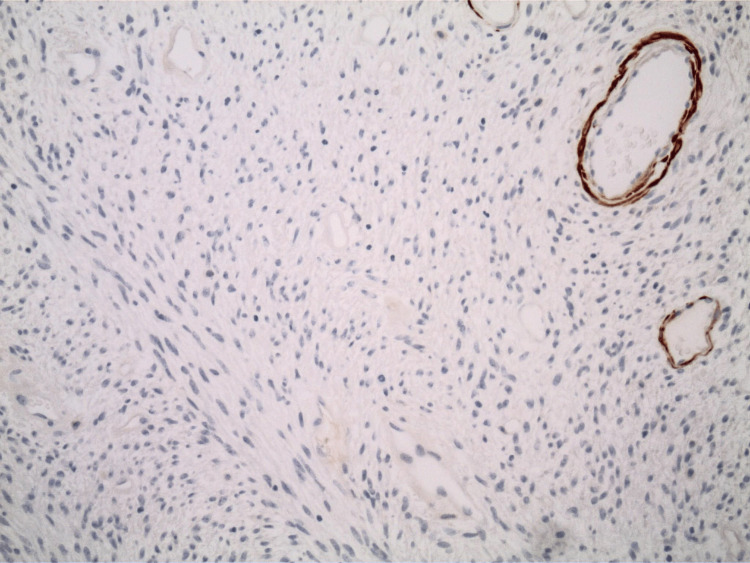
100x magnification of solitary fibrous tumor with immunohistochemical staining that is negative for desmin.

**Figure 12:**
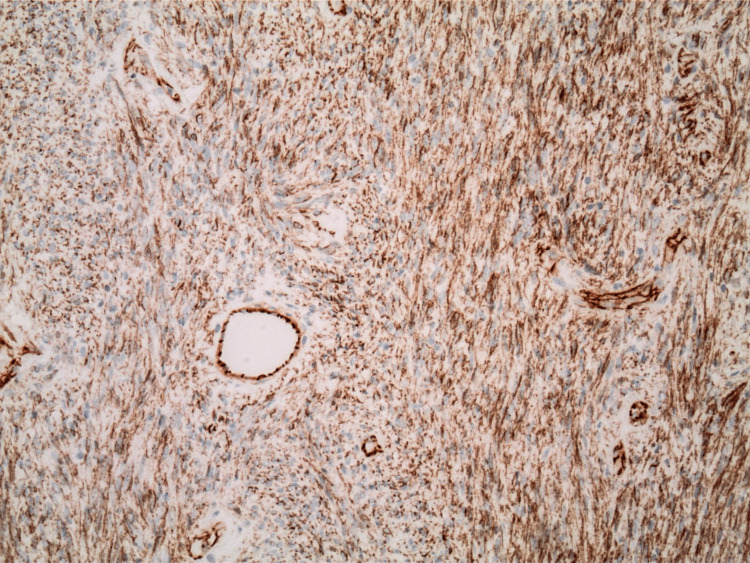
100x magnification of solitary fibrous tumor with immunohistochemical staining that is negative for beta-catenin.

**Figure 13:**
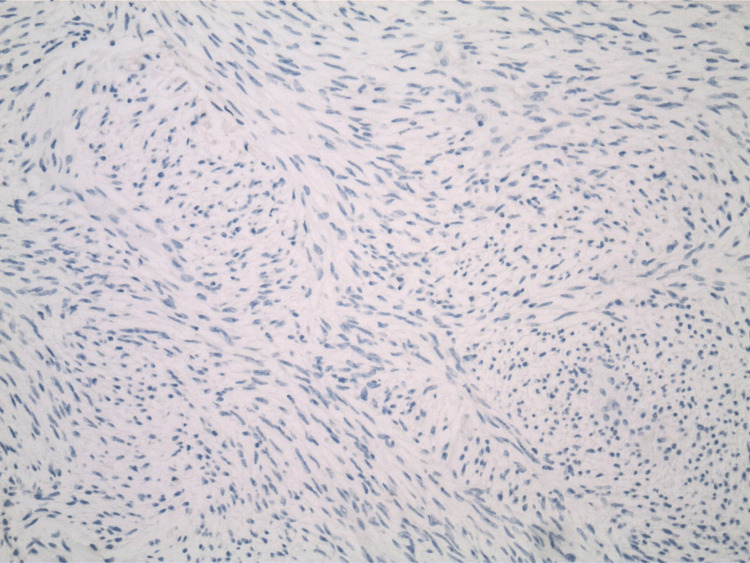
100x magnification of solitary fibrous tumor with immunohistochemical staining that is negative for pan-cytokeratin.

**Figure 14:**
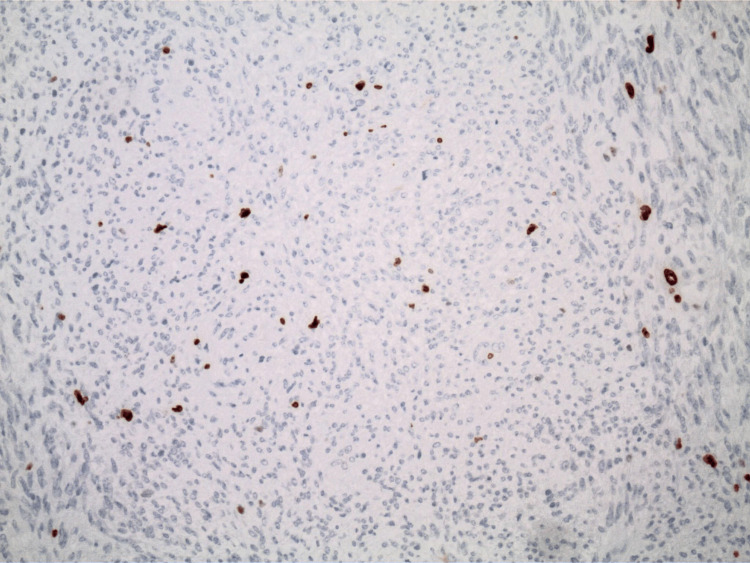
100x magnification of solitary fibrous tumor with immunohistochemical staining that demonstrates a Ki-67 index less than 5%.

The pathology report indicated that the mass was a solitary fibrous tumor. The histologic section demonstrated a moderately cellular spindle cell tumor in collagenous stroma with a prominent dilated staghorn-type vasculature (Figure 2). Furthermore, a mitotic rate of 2 mitoses per 10 high-power fields was identified (Figure 3), along with tumor necrosis for approximately 10-20% of the mass (Figure 4). Immunohistochemical studies showed that the tumor cells were positive for CD34 (Figure 5), positive for vimentin (Figure 6), and positive for STAT6 (Figure 7). Meanwhile, the tumor cells were negative for SOX10 (Figure 8), negative for S100 (Figure 9), negative for SMA (Figure 10), negative for desmin (Figure 11), negative for beta-catenin (Figure 12), and negative for pancytokeratin (Figure 13). Lastly, the Ki-67 mitotic index was less than 5% (Figure 14).

 

## Discussion

The majority of SFTs are discovered incidentally on chest imaging, because they are usually asymptomatic unless they grow to a size that can cause mass effect on other organs [1]. Contrast-enhanced computed tomography (CT) imaging generally shows a well-circumscribed, lobulated, hypervascular tumor, which may have areas of necrosis if the tumor is large in size [1]. Meanwhile, T2-weighted magnetic resonance imaging (MRI) shows a heterogenous well-circumscribed mass with hypointense signals where the tumor has a lot of fibrous content and large bright areas that may indicate hemorrhage, cystic degeneration, or central necrosis [1,3]. Nonetheless, imaging is not sufficient to diagnose an SFT, and therefore, biopsy is recommended for further analysis [1,3].

Grossly, most SFTs are well-circumscribed with a smooth surface and a lobulated shape at 7-10 cm in size, although SFTs have been known to range anywhere between 1 and 40 cm in size [1]. Histologically, SFTs show a “patternless” architecture of thick collagen bands, spindle tumor cells with scant cytoplasm and indistinct cell borders, and “staghorn” blood vessels in a hemangiopericytoma-like vascular pattern [1,3,5]. SFT immunohistochemistry (IHC) is relatively nonspecific and consistently positive for CD34 while variably positive for CD99, vimentin, BCL2, nuclear beta-catenin, and epithelial membrane antigen (EMA) [1,5]. In particular, vimentin staining can help confirm the mesenchymal origin of SFTs [6]. Furthermore, SFT IHC is negative for cytokeratin, S-100, desmin, and alpha-smooth muscle keratin [3].

Molecular genetic analysis is the most helpful tool for differentiating SFTs from other soft tissue tumors, as the NAB2-STAT6 gene fusion has been proven highly sensitive and specific for SFTs, being present in 91% of SFTs [1]. The NAB2-STAT6 fusion gene results from an inversion at the 12q13.3 locus of chromosome 12, which can be detected by reverse transcription polymerase chain reaction (PCR) [5,7]. NAB2 is a transcriptional repressor that is associated with the early growth response (EGR)-mediated pathways, which is mediated by the nucleosome remodeling and deacetylase complex and consists of an N-terminal EGR1 binding domain (EBD), a C-terminal transcriptional repressor domain (RD), and NAB conserved regions 1 and 2 (NCD1 and NCD2) [1,2,5,7,8]. Meanwhile, STAT6 is a transcription factor with a central role in interleukin 4 (IL4)-mediated biological responses that consists of a DNA-binding domain (DBD), a C-terminal transcriptional activation domain (TAD), and an SH2 domain [5,7,8]. When NAB2 and STAT6 are fused together, the RD of NAB2 is truncated and fused together to the TAD of STAT6, which leads to the constitutive activation of EGR1-mediated transcription, thereby allowing for an increase in cellular proliferation and neoplastic progression [5,7,8]. NAB2-STAT6 have a large number of fusion variants, of which the most common is the NAB2ex4-STAT6ex2/3 fusion variant that corresponds mainly with pleuropulmonary SFTs in older patients at a median age of 69 years old [2,9]. Nonetheless, studies have found that NAB2ex6-STAT6ex16/17 is the most common fusion variant in extrapleural locations with an earlier median age of 47 years old, as well as a more aggressive clinical behavior despite being significantly smaller in size than the STAT6ex2/3 variants [2,9]. In general, the NAB2ex6-STAT6ex16/17 SFT shows a higher mitotic count, higher cellularity, less hyalinization, and more staghorn-like branching vessels than the STAT6ex2/3 variant [2].

Although 12-22% of SFTs are malignant, it can be difficult to predict which SFTs will follow a metastatic course based upon histologic features alone [4]. Nonetheless, the World Health Organization accepts the use of a four-variable risk stratification model developed by Demicco et al., which determines the risk of SFT metastasis based upon age, tumor size, mitotic count (per 10 high-power fields), and the degree of tumor necrosis [10]. According to this model, the patient of this case was determined to have an intermediate risk of SFT metastasis, due to having an age of 68 years old, a tumor size of 12 cm, a mitotic count of 2 mitoses per 10 high-power fields, and tumor necrosis of 10-20%, as shown in Table 1.

**Table 1:** Assessment of the patient's SFT metastatic risk according to the four-variable risk stratification model developed by Demicco et al. SFT: solitary fibrous tumor

Four-variable SFT metastatic risk stratification model developed by Demicco et al.			Patient's SFT metastatic risk assessment according to model
Risk factor		Score	Assessment of the patient's risk factors
Age (years)	<55	0	68 years old (score of 1)
	≥55	1	
Tumor size (cm)	0 to <5	0	Tumor size at 12 cm (score of 2)
	5 to <10	1	
	10 to <15	2	
	>15	3	
Mitotic count (per 10 high-power fields)	0	0	2 mitoses per 10 high-power fields (score of 1)
	1 to 3	1	
	≥4	2	
Necrosis (%)	<10	0	10-20% tumor necrosis (score of 1)
	≥10	1	
SFT metastatic risk		Total score	Assessment of the patient's SFT metastatic risk
Low		0-3	Intermediate risk of SFT metastasis (total score of 5)
Intermediate		4-5	
High		6-7	

The management of SFTs involves a wide surgical resection of the tumor while preserving surrounding organs and structures, with 10-year survival rates between 54% and 89% after complete surgical resection with clear margins [1,11]. Post-operative surveillance involves imaging the primary tumor site every three to four months for the first two years after the surgery and every six months for years two through five after the surgery [12]. Once five years have been reached, imaging the primary tumor site is no longer necessary, as local recurrence of the SFT at that point would be unusual [12]. Although local recurrence has been shown to occur at a rate of 8%, neither radiation therapy nor chemotherapy after resection have yet to show benefit and, therefore, are not currently recommended [1].

## Conclusions

SFTs are rare tumors of mesenchymal origin that are often pleural, benign, and asymptomatic, although some SFTs have been known to be extrapleural and malignant and cause mass effect on other organs once the SFT has reached a certain size. While SFTs have been known to appear incidentally on CT and MRI imaging, biopsy remains necessary to confirm diagnosis, as there are certain histological, immunohistochemical, and molecular genetic features that are unique to SFTs. Once a diagnosis has been confirmed, the risk of SFT metastasis can be determined using the four-variable risk stratification model developed by Demicco et al. Management of an SFT is focused upon the wide resection of the tumor and continued follow-up with the patient, in order to ensure that there is no local recurrence of the SFT.

Learning objectives for this case include (I) discerning the risk factors for SFTs; (II) recognizing how SFTs typically presents clinically and on imaging; (III) identifying the gross, histological, immunohistochemical, and molecular genetic features of SFTs; (IV) understanding how to predict the risk of SFT metastasis; and (V) knowing the treatment of choice for SFTs.
